# Step-Index (Semi-Immersed) Model for Photonic Nanojet and Experimental Characterization via Near-Field Optical Microscopy with Microcylinder

**DOI:** 10.3390/nano13061033

**Published:** 2023-03-13

**Authors:** Tal Elbaz, Ankit Chauhan, Aviran Halstuch, Gil Shalev, Alina Karabchevsky

**Affiliations:** School of Electrical and Computer Engineering, Ben-Gurion University of the Negev, Beer-Sheva 8410501, Israel

**Keywords:** photonic hook, photonic nano jet, optical fiber, near-field

## Abstract

Experimental limitations such as design complexity and low optical throughput have prevented photonic nanojet (PNJ) and photonic hook (PH) measurements from demonstrating and characterizing the implementation of narrow intense electromagnetic beams generated from dielectric microelements with circular symmetry. Near-fields optical microscopy can mitigate these limitations and still present a capability of detecting a highly localized electromagnetic beam for applications in step-index media. Here we model a localized PNJ and PH formation in step-index media. We show that despite negligible refractive index contrast between the water ($n_{water}=1.33$) and silica microcylinder (∼1.1), a formation of PNJ and PH is observed with equivalent performance compared to that of silica microcylinder embedded in air ($n_{air}=1$). This model features a practical fiber source and silica microcylinder as an auxiliary structure. Simultaneously, we performed experimental characterization of a photonic nanojet generated from an optical fiber and studied the resulting near-fields. Our electromagnetic simulation results are in good agreement with the experimental ones, demonstrating a full width at half maximum (FHWM) with a relative error of 0.64%. This system will make fiber-based nanojet realization and characterization accessible and practical for optics and laser engineering applications, super-resolution imaging, and nanolithography.

## 1. Introduction

Microelements made of dielectric materials with circular cross section, such as microspheres and microcylinders, have shown interesting optical properties; they can confine light inside the cavity, forming whispering gallery modes (WGMs), or focus the light in their proximity, forming photonic nanojets (PNJs) and phjotonic nanohooks (PH) [1,2,3].

PNJs are narrow intense electromagnetic beams emerging from the ’shadow-side’ surface of a plane-wave-illuminated dielectric microcylinder or microsphere (with a diameter greater than the illuminating wavelength) propagating into the surrounding medium. Those non-resonant, localized, high intensity, and low divergence beams [4] characterized by narrow and spatially highly confined beams, were first demonstrated in 2004 by Chen et al. [5]. They are usually generated at the shadow side of the auxiliary structure [6] usually illuminated by continuous wave (CW) or by pulse illumination [7]. This field gives rise to interference effects of the optical field [8]. By partially blocking the incident light, so its symmetry breaks, the convergence of the focusing field demonstrates a curved PNJ, known as a photonic hook (PH) [9,10,11,12]. Depending on deformation, it has unique properties of a tilt angle [13]. The distinctive features of the PH are the transverse size, and curvature radius of the beam [14]. By varying the part of the cylinder block with a mask, one can tune the angle of the hook. Another method is breaking the symmetry of the focusing object itself, locally changing the refractive index of a cylinder [15], or fabricating a non-symmetric structure [9].

According to the Lorenz-Mie theory of plane-wave scattering by a spherical particle [16,17], the optical fields inside and outside the dielectric microelement are subject to the light wave due to the curvature of the particle surface and acting as a refractive microlens [18,19]. The photonic nanojet phenomenon possesses unique properties: focal distance, longitudinal length, lateral full width at half-maximum (FWHM), potentially going beyond the diffraction limit, and maximum intensity [20]. Due to its unique properties, it can be utilized for super-resolution imaging [21], laser writing [22], optical manipulation and trapping [9,23], photolithography at the nanoscale [2,24,25], backscattering enhancement [26,27], Raman scattering [28], ultra-high transmission optical waveguide [29], single nanoparticle or molecular detection [30,31], quantum dots writing [32] and biological applications using nanoparticles [3,33]. For achieving PNJ and PH formation, a high refractive index contrast (RIC) between dielectric materials and their surroundings is required. The refractive index contrast (RIC) between the microcylinder and its surrounding medium and the microcylinder dimensions is critical in optimizing the key parameters: focal length, intensity, FWHM, and length [25,34,35]. The contrast between particles and the environment is low for specific applications, such as manipulating cells. These high-index materials, required to create a high enough refractive index contrast between the dielectric material and surroundings, might cause mechanical and photothermal damage to biological cells [25,36]. Here we show that, by using a step-index medium, one can avoid using high-index microcylinders. In addition, most auxiliary structures are based on nanoscale objects that require a complex and costly fabrication process [34,37]. As reported in our previous work [13], using a microcylinder perpendicular to the substrate is an elegant way to generate a photonic nanojet and photonic hook. In continuation, this work proposes a commercial single-mode optical silica fiber as a dielectric microcylinder to be utilized as a common non-complicated auxiliary structure for PNJ formation. As the PNJ is characterized by a narrow FWHM very close to the diffraction limit and even capable of going beyond it [20], it becomes challenging to perform the transverse dimension imaging utilizing optical microscopy [38]. Furthermore, due to its formation in the near field [20], we propose to utilize a near-field scanning optical microscopy (NSOM), considering that the nanoscale light management can overcome these limitations [39]. The NSOM belongs to the family of scanning probe technologies that allow the collection of diverse surface information. A prominent member of this family is atomic force microscopy (AFM), used to measure the morphology/topography of nanostructured samples [40,41]. This is based on the proximal-probe technique that can perform imaging of near-field optical signals with subwavelength resolution beyond the diffraction limit [5,6]. By collecting laser light into the hollow fiber tip, AFM can fabricate arbitrary-shaped nanopatterns. The resolving power of the NSOM depends on tip opening size [42]. In this work, we propose a configuration of a step-index medium, using a common material for auxiliary structure, which can potentially offer the design of an elegant, non-complicated system for optomechanical manipulations, particularly for applications in an aqueous environment. Additionally, we report the approach to generating and characterizing a PNJ out of an intensity profile measurement with a conventional NSOM system for nano-resolution and near-field collection.

## 2. Materials and Methods

### 2.1. Numerical Simulation

**Figure 1:**
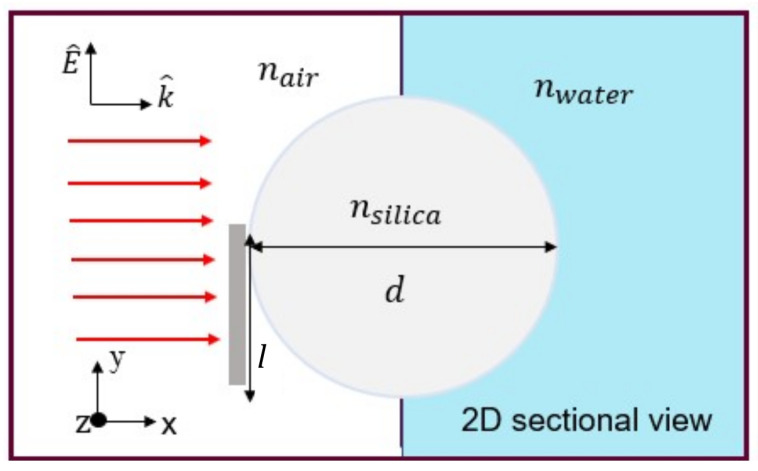
Schematic illustration of the numerical model for PNJ & PH formation in a step-index medium with 2D cross-section of auxiliary structure made of silica. Plane-wave illuminates a mask in air, in the direction indicated by vector *k*. Mask is partially blocking the cylinder. The generated beam is created in water.

Figure 1 illustrates a proposed schematic of our model. The dielectric microcylinder of a circular cross-section made of silica with a diameter (*d*), is illuminated by an incident linearly-polarized continuous plane wave in visible. We built a model in COMSOL Multiphysics 5.6 platform which is based on finite element analysis (FEM), a method applicable to wave optics module computational approaches in nano-optics and near-field optics geometries [6,43]. The perfectly matched layer (PML) absorbing boundary condition was set to all exterior boundaries of the simulation. The PML layer width is equal to 7 μm. At the microcylinder shadow side, additional space with a width of ∼70 μm was added to visualize and study the resulting photonic jet and photonic hook. A free triangular mesh was used for the free-space regions with a maximum element size of λ/(5n). Additionally, a free triangular mesh with a maximum element size of λ/(6n) was set to the microcylinder to obtain results of satisfactory resolution. An aluminum (Al) screen (mask) with a width of 0.1 μm and a maximum mesh size equal to 0.1 μm partially blocks the incident light as an essential condition for the formation of the photonic hook. The aluminum mask has the complex refractive index of $n_{\mathrm{Al}}=1.17+i6.5$ [44], corresponding to the wavelength of λ=637 nm, and is located at two different heights (*l*) concerning the microcylinder diameter: (1) l=0d and (2) l=0.25d for symmetry (PNJ) and non-symmetry (PH) illumination, respectively. For simplicity in the numerical calculation, the cylindrical structure is considered a 2D cross-section due to its physical structure; the microcylinder length is much larger than the diameter and is uniform, so the changes throughout the 3rd direction can be overlooked. It is set as transparent with refractive index of $n_{silica}=1.46$ (can be fused silica) and background of $n_{air}=1$. The initial intensity of the illuminated light in the *x*-direction is set to 1 $\mu W/m^{2}$. In order to examine the optical response of the proposed step-index medium model, the incident light illuminates the microcylinder, partially blocked by a mask, in two different configurations: (1) from air into air, and (2) from air into water with a refractive index of $n_{air}=1$ and $n_{water}=1.33$ respectively, as shown in Figure 1. Furthermore, the microcylinder diameter is changed between 4 and 12 μm to examine its effect on the proposed model. The resulting data were analyzed with MATLAB computing platform, academic license version R2021a.

Further, to design an optical system and to explore the PNJ effect and analyze the obtained experimental results, we built another model to predict the photonic nanojet properties (see Figure 6). A perfectly matched layer (PML) bounded the 2D simulation at all sides. The mesh was explored to ensure the accuracy of the calculated results. Here, The microcylinder has a refractive index of $n_{silica}=1.457$, corresponding to fused silica ($SiO_{2}$) in the wavelength of 637 nm in the visible spectral range [45]. The background is of $n_{air}=1$. The initial intensity of the illuminated light kept the same as mentioned above.

### 2.2. Fiber Preparation

**Figure 2:**
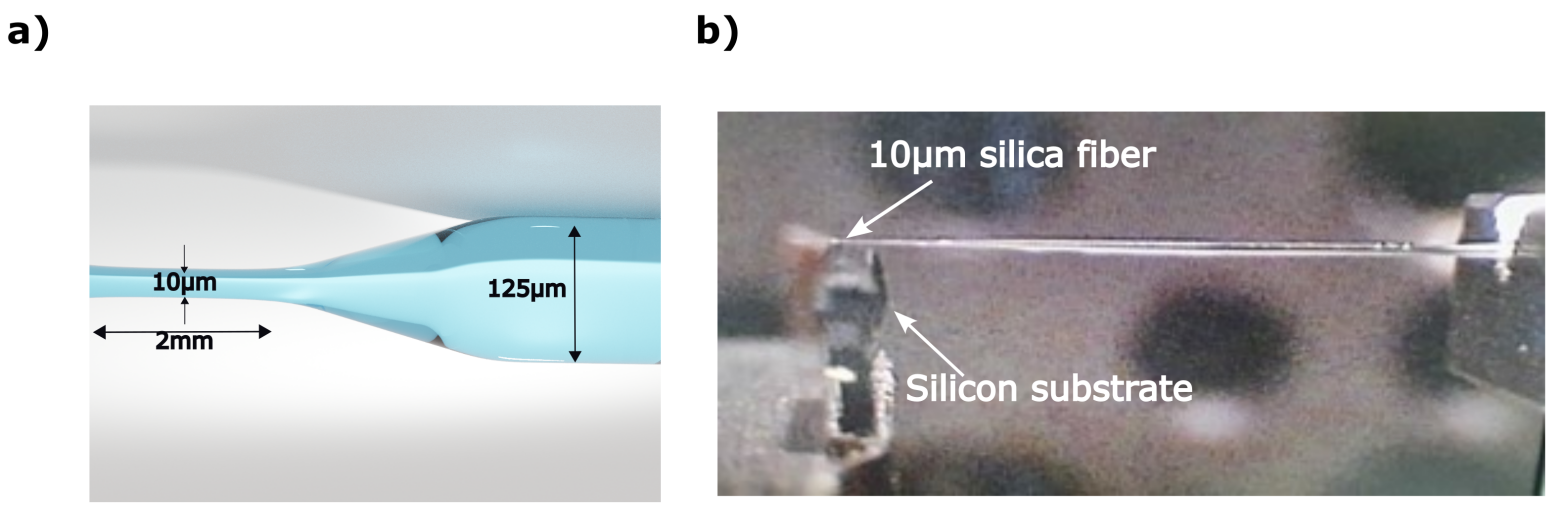
Optical fiber tapering for the experiment: (**a**) Rendered fiber architecture with obtained dimensions. (**b**) Photograph of the tapered fiber of adiabatic tapering, captured during gluing of the fiber onto the silicon substrate.

The optical fiber we used as an auxiliary structure is commercial silica single-mode optical fiber tapered down to 10 μm out of 125 μm cladding diameter with an accuracy of 0.25 μm. The fiber was pulled using Vytran GPX-3400 glass processor, with a velocity of ∼1 mm/s, which caused it to gradually taper off, as demonstrated in Figure 2a. Next, the fiber was glued to the silicon wafer to form a reflection and enable the imaging with an NSOM probe, as shown in Figure 2b.

Figure 2a shows a rendering of the tapered fiber, while Figure 2b shows the fabricated tapered fiber glued to the silicon substrate.

### 2.3. Experimental Setup

**Figure 3:**
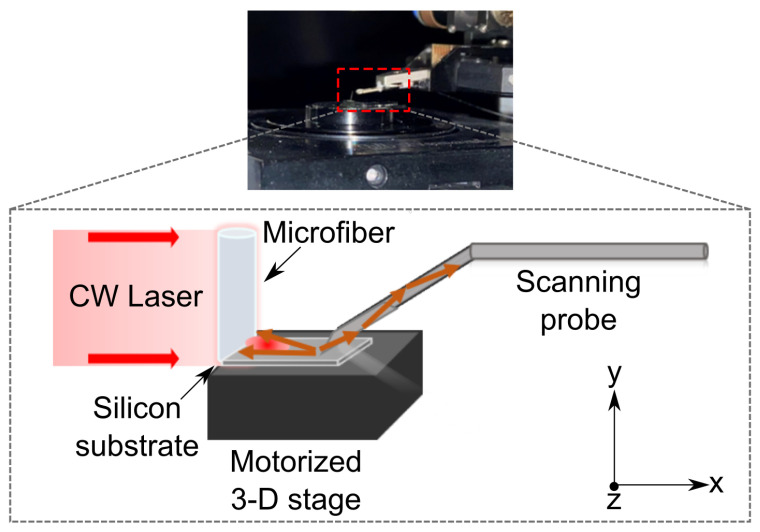
Experimental setup: (**top**) the photograph of the fiber on the substrate placed on the motorized stage while the NSOM probe scans the near-fields; (**bottom**) schematics of the experimental setup with the microfiber illuminated by the CW light and scanned via the NSOM probe.

To examine the PNJ phenomenon in practice, we performed an experiment that combines NSOM measurement to overcome the magnification limitation. In the current study, we employ near-field scanning optical microscopy (NSOM), to provide an experimental characterization of PNJs formation by illuminating a silica microfiber. Although NSOM has been used extensively to study the near-field behavior of dielectric and metallic arrays and isolated subwavelength structures [46,47,48,49,50], to our knowledge, it has never been utilized in PNJ studies for characterization out of intensity profiling. Here, we employed the NSOM to measure the light intensity distribution obtained on the silicon surface to measure the near-fields (i.e., the formed PNJ). Unlike conventional NSOM measurements, the laser light that we used as the light source is not the built-in one of the NSOM system but rather an external light placed next to the NSOM. The experiment was performed with a non-polarized collimated laser beam at wavelength λ=637nm applied on a 10 μm diameter silica optical fiber. The laser is propagating in the *x*-axis attenuated by a variable density filter to avoid the saturation of the NSOM photodiode. The sample of the optical microfiber was located on the NSOM piezoelectric stage. The NSOM probe was located at less than 0.2 μm from the microfiber for the scanning and detection. The probe moves approximately a few tens of nm above the surface along the *y*-direction on set oscillation frequency and based on feedback during the scan. The NSOM system used for collecting the near-field photons of PNJs is MultiView-4000 by Nanonics. The count rate of the photodiode was set to 100 kHz, and the quantum efficiency of the detecting photodiode was 0.7. The experimental setup of a tapered optical fiber glued to a silicon substrate and scanned by an NSOM probe is shown in Figure 3. Note: Using the same experimental approach one can measure the PH in the experiment. For this partially blocking light mask needs to be positioned before the fiber.

## 3. Results

### 3.1. Numerical results

**Figure 4:**
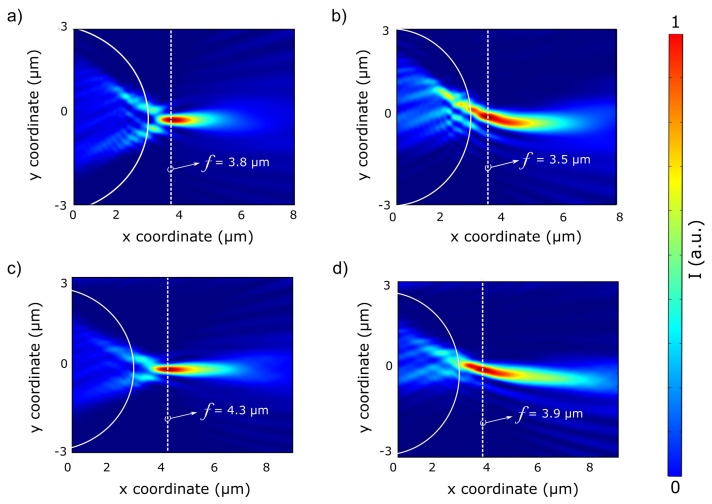
Normalized distribution of the Poynting vector $S_{x}$ via a 6 μm diameter silica microcylinder illuminated by a 637 nm continuous plane-wave in the *x*-direction. Solid white lines represent the microcylinder boundary and dashed white lines represent the focal point *f*. (**a**) PNJ formation in air. (**b**) PH formation in air. The mask blocks 25% of the illuminating light. (**c**) PNJ formation in water while the cylliner is illuminated from the air. (**d**) PNJ formation in water while light is illuminated from the air and the mask blocks 25% of the illuminating light.

The simulations consist of a numerical model that examines the feasibility of PNJ and PH through a silica microcylinder at low refractive index contrast (RIC) conditions. The RIC between silica and the surrounding medium (i.e., refractive index ratio $n_{silica}/n_{water}$) is equal to 1.1, which is relatively low compared to 1.46 between silica and air for a wavelength in the VIS range of 637 nm. Figure 4 presents the numerical simulation for PNJ and PH formation. It shows the Poynting vector $S_{x}$ of a 6 μm diameter silica microcylinder and an aluminum mask deposited at (1) l=0d, (2) l=0.25d in air and air-water surroundings. The illuminating beam is a 637 nm continuous plane wave in the *x*-direction with an initial intensity of 1.0 mW/m${}^{2}$. The focal length (*f*) is measured from the center of the cylinder cross-section located the axes’ origin. A comparison between the normalized distribution of the Poynting vector in the *x*-direction of the two different surroundings: (1) air (Figure 4a,b), (2) air-water (Figure 4c,d) shows that using the step-index configuration (i.e., silica microcylinder illuminated from air into water) results in PNJ and PH performances at the near field, despite the differences between RIC. The values for PNJ and PH characterized parameters are summarized in Table 1.

Note: A convergence plot, shown in the Supplementary File, shows the mesh sizes that were found to be optimal. For this we carried out a numerical study in which we explored the influence of fine and coarse mesh on the improvement of the results. The computational time which accounts for mesh size was also studied.

Table 1 compares the main parameters that characterize the PNJ and PH between the two surroundings, as demonstrated in Figure 4 for two different heights of the aluminum screen.

One can observe a clear trend of increasing focal length and longitudinal size of the PNJ and PH as the RIC ratio between the microcylinder (silica) and its surrounding decrease. The main reason for these differences is water’s presence, increasing field scattering. Moreover, there is only a slight difference in maximum intensity at the focal point between the two surroundings, despite the considerable difference in RIC. By examining the PH tilt angle, results show a smaller tilt angle for a step-index medium due to different phase velocities in different mediums resulting in a slightly different wavefront deformation. Considering the obtained FWHM results of PNJs, both surroundings demonstrate an FWHM that goes beyond the diffraction limit, below half of the wavelength. Generally, the values for all the characterized parameters: focal length, tilt angle, maximum intensity, FWHM, and longitudinal size have the same order of magnitude. The differences are mainly due to the different refractive indexes of the two surroundings, leading to differences in scattering. For example, the longitudinal size is more prominent in water due to increased scattering as the incident light enters the water. The significant outcome of the results is that despite negligible RIC between water and silica (∼1.1) [25,35], a formation of PNJ and PH is observed with equivalent performance compared to that of air (n = 1). Furthermore, both configurations demonstrate the capability for super-resolution when considering the PNJ performance. Hence, a model of a step-index medium enables the diversity of the dielectric materials that can be used as an auxiliary structure, particularly microcylinder, in a liquid surrounding.

**Figure 5:**
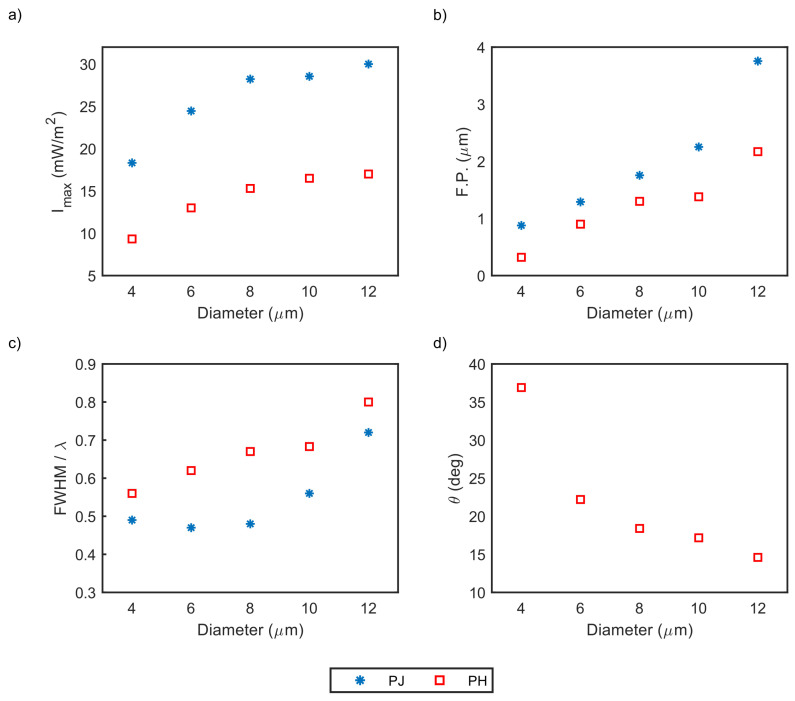
PNJ and PH characterized parameters as a function of the silica microcylinder diameter, which is illuminated by a 637 nm continuous plane-wave in the *x*-direction. (**a**) Maximum intensity for PNJ (blue) and PH (red) (**b**) Focal point (F.P.) for PNJ (blue) and PH (red) measured from the center of the microcylinder that is located on axes-origin (**c**) FWHM for PNJ (blue) and PH (red) related to the incident wavelength (λ) (**d**) PH tilt angle (θ) in degrees.

In order to determine the microcylinder diameter effect for the step-index model, the diameter impact was examined in terms of super-resolution (FWHM), maximum intensity, focal point (F.P), and tilt angle (PH only) for varying diameter sizes between 4 and 12 μm. Figure 5 concludes the characterized parameters of PNJ and PH: maximum intensity, focal point (F.P.), FWHM, and tilt angle as a function of the microcylinder diameter. The focal point values are present as the focal distance—the difference between the microcylinder boundary and the focal point.

One can see from Figure 5 clear trends for PNJ and PH key parameters as the microcylinder diameter changes. As expected, the maximum intensity per surface unit increases as the microcylinder diameter increases due to the greater internal reflection that results from the inner side of the shadow surface of the microcylinder of a larger diameter compared to the microcylinder with a smaller diameter. The focal point also increases as the microcylinder diameter increases, namely, the radius of curvature increases. Moreover, the FWHM in terms of the incident wavelength (λ) increases as the diameter increases and demonstrates a sub-diffraction effect (smaller than half of the wavelength) for PNJ configuration only. The tilt angle (θ), is relevant only for PH configuration and decreases as the microcylinder diameter increases. Considering the FWHM performances essential for super-resolution imaging, a 6 μm diameter represents the best performance. The relation between the interference of the incident beam with the scattered field can reach the strong subwavelength confinement. It happens when the diameter of the microcylinder has a dimension of about ten times the wavelength [18]. As the diameter increases relative to the incident wavelength, the scattering angle extends, resulting in a broader FWHM. The maximum intensity has the same order of magnitude for different diameters without significant differences. However, there is a significant difference between PNJ and PH in maximum intensity. The PNJ maximum intensity is approximately twice as great as the intensity of PH due to extra interactions between light and microcylinder.

### 3.2. Experimental Characterization Results

**Figure 6:**
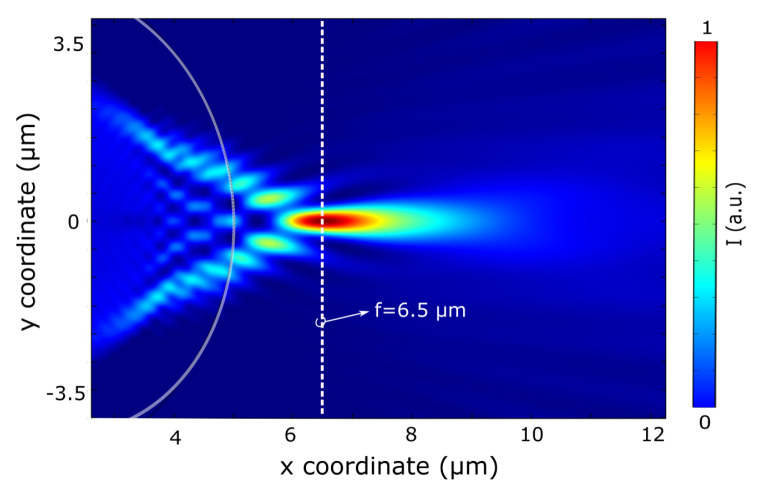
Calculated intensity of PNJ creation via a 10 μm diameter silica microcylinder illuminated by a 637 nm continuous plane wave in the *x*-direction.

The numerical simulation results shown in Figure 6 represent a PNJ formation on the shadow side of the 10 μm diameter silica microcylinder, illuminated by a linear-polarized CW with a wavelength of λ=637 nm. The PNJ focal point is located along the optical axis at 6.5 μm. This point is measured from the center of the cylinder as in ref. [4,51], which is equal to the cylinder radius. Figure 6 shows the normalized Poynting vector distribution of the microcylinder cross-section with well-pronounced jet formation.

**Figure 7:**
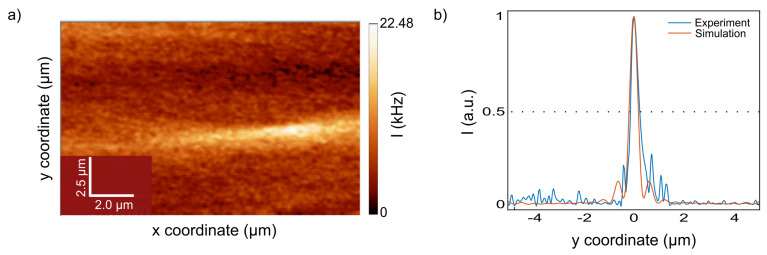
Experimental results were obtained with an NSOM scan of a 10 μm diameter Silica microcylinder illuminated by a 637 nm continuous plane wave in the *x*-direction. (**a**) Scanned image in photon counts units using the photodiode. (**b**) The normalized intensity distribution was extracted from the NSOM image (blue) compared to the calculated intensity (orange) for the simulated 10 μm diameter Silica microcylinder. The dashed black line indicates the FWHM.

We implemented experimentally the concept shown in the numerical model that we built using the Finite Element method (FEM). The image detected near-field by the NSOM probe is shown in Figure 7a. The photon counts were detected via an NSOM probe during continuous scanning. One can observe a narrow optical jet-like effect generated by an external collimated laser beam illuminating optical microfiber [52], which is positioned out-of-plane, compared to the substrate. This jet-like image is characterized by a focal spot, as seen from the longitudinal profile. Figure 7b shows the intensity distribution of the experimentally observed photonic jet-like focused beam as compared to the numerical calculation.

The main characteristics of the photonic jet-like beam from numerical and experimental results, both for a 10 μm diameter Silica microcylinder, are summarized and shown in Table 2 for comparison.

It can be observed from Table 2 that the focal length, obtained experimentally and numerically, is 6.7 and 6.5 μm, respectively, with an accuracy of 200 nm and a relative error of 3.0%. The measurements were performed at the focal point, measured as the distance from the center of the fiber that is considered equal to 5 μm which is the fiber radius. Maximum intensity was measured as the intensity at the focal point of the photonic jet-like beam, and intensity enhancement was measured as compared to the input intensity of the illuminating laser beam. The maximum intensity at the focus of the simulated photonic jet-like beam is 25.6 $\mu W/m^{2}$ compared to an initial illuminated intensity of 1 $\mu W/m^{2}$; hence, the theoretical improvement is 25.6. The experiment’s results demonstrated an improvement of approximately 14 times the initial illumination intensity after consideration of the quantum efficiency of the detection photodiode.

We note that since the properties of light may affect the spatial characteristics of PNJ [53], the non-polarized laser light source in the experiment vs. the linearly polarized light source in the simulation may result in these differences. From the intensity distribution at the focal point in Figure 7b, we calculated the FWHM and compared it to the simulation results. The experimental results are in good agreement with numerical simulation, as shown in Figure 7b, with a relative error of 0.64%. Moreover, the calculated longitudinal size is 2.5 and 2.4 μm for simulation and experiment, respectively, with a relative error of 4.0%. The effect of narrowing the photonic nanojet exists only near the particle surface at distances of about a quarter of the incident wavelength. Here we aimed at making the mesoscale structure as small as possible to obtain the focal spot located at the surface since the PNJ characteristics increase with the radius of the auxiliary structure [19,54,55]. Accordingly, despite the known potential of the PNJ of going beyond the diffraction limit, this could be achieved with a diameter of 10 μm of microfiber as a proof of concept characterization of focused jet-like beams. The freedom to illuminate in an asymmetric off-axis laser can be tuned for a photonic hook formation as a potential for optical manipulation on 3-D objects [23,37,56]. A further advantage of this system is the ability of the NSOM to perform the scan in water. This function can enable the performance of PNJ in aqueous and step-index environments for biological applications [57] and in both academic research and industrial R&D laboratories.

## 4. Conclusions

Recent advances in nanotechnology and imaging techniques based on near-field scanning probes, molecular fluorescence, microscale, and nanoscale solid immersion lenses, and metamaterials have been proposed in recent years to detect the generation of highly localized electromagnetic beams such as photonic nanojet and photonics nanohook, and more, to overcome the diffraction limit. However, the design complexity, the need for high-index auxiliary structures to operate in an aqueous environment of biological cells, and low optical throughput time have impeded their use to some extent. We present a step-index model for photonic jet formation with full width at half-maximum (FWHM), capable of going beyond the diffraction limit despite low contrast between aqueous medium (∼1.33) and silica auxiliary structures (∼1.46). These high-index materials, required to create a high enough refractive index contrast between the dielectric material and surroundings, might cause mechanical and photothermal damage to biological cells. The significant outcome of the results is that despite negligible refractive index contrast between water and silica (∼1.1), a formation of PNJ and PH is observed and demonstrates equivalent performance compared to silica microcylinder embedded in the air (n = 1). Furthermore, both configurations demonstrate super-resolution capability when considering the PNJ performance. We also show a method to characterize the photonic nanojet. We formed the photonic nanojet via external laser light and detected the near-fields with near-field scanning probes. For this, we fabricated the auxiliary structure of a circular cross-section on the high-index substrate. Dielectric microcyllinder was obtained by tapering the silica fiber’s core and cladding locally and then gluing it to the silicon substrate. The near fields were collected with the near-field scanning optical microscope. Here we show that one can avoid using high-index materials by using a step-index medium. Our results showed a good agreement between the calculated and measured intensity distribution at the focal point. This method may be useful for any imaging-starved application, such as for nondestructive real-time imaging of specimens at a resolution beyond the reach of human eyes at low-index contrast environments such as physiological media. That may provide a new ultramicroscopy technique for using visible light to detect and image nanoparticles such as proteins, viral particles, and even single molecules; and monitoring molecular synthesis and aggregation processes of importance in many areas of biology, chemistry, material sciences, and tissue engineering. More generally, this technique demonstrates a practical, scalable, and robust approach to obtaining optical information at the nanoscale level.

The proposed characterization method allows to generate and visualize PNJ while employing the advantages of scanning near-fields.

## Figures and Tables

**Table 1:** Photonic Jet formation via illumination on 6 μm silica microcylinder in air. vs. air-water.

Medium	Screen Height [μm]	Focal Length [μm]	Tilt Angle θ $\left[{}^{\circ }\right]$	Maximum Intensity [W/m${}^{2}$]	FWHM	Longitudinal Size [nm]
Air	0d	3.8	0.0	$18.5\times 10^{-3}$	0.48 λ	5.3
	0.25d	3.5	45.0	$12.6\times 10^{-3}$	0.54 λ	6.0
Air-water	0d	4.3	0.0	$18.6\times 10^{-3}$	0.47 λ	7.0
	0.25d	3.9	30.0	$12.9\times 10^{-3}$	0.62 λ	9.0

**Table 2:** Photonic jet-like beam formation via illumination on 10 μm silica microcylinder in free space.

Method	Focal Length (μm)	Intensity	FWHM	Longitudinal Size (μm)
Simulation	6.5	25.6	0.584 λ	2.5
Experiment	6.7	14.3	0.578 λ	2.4

## Data Availability

Data underlying the results presented in this paper are not publicly available at this time but may be obtained from the authors upon reasonable request.

## Supplementary Materials

The following supporting information can be downloaded at: https://www.mdpi.com/article/10.3390/nano13061033/s1, Figure S1: Numerical simulation convergence test at cross-section—convergence test for four mesh sizes: λ/3*n*, λ/6*n*, λ/9*n* and λ/18*n* at cross section of *y* = 0. (a) Intensity along the *x*-direction of the numerical model. (b) A logarithmic view of intensity values; Figure S2: Calculated photonic nanojet characterized parameter as a function of 4 different mesh sizes: λ/3*n*, λ/6*n*, λ/9*n* & λ/18*n*. (a) FWHM in terms of incident wavelength (λ), (b) Maximum intensity (mW/m^2^), (c) Focal point (F.P.) and (d) relative error calculation between the mesh presented in the thesis and smaller mesh.
