# Supplementary material for: Step-Index (Semi-Immersed) Model for Photonic Nanojet and Experimental Characterization via Near-Field Optical Microscopy with Microcylinder
Source: Nanomaterials (Basel). 2023 Mar 13;13(6):1033. doi: 10.3390/nano13061033 (PMC10052061; doi:10.3390/nano13061033)
Supplement: Supplementary file 1 [file nanomaterials-13-01033-s001.zip › nanomaterials-2193089-supplementary.pdf]

Supplementary Information

Step-Index (Semi-Immersed) Model for Photonic Nanojet and Experimental Characterization via Near-Field Optical Microscopy with Microcylinder

Tal Elbaz, Ankit Chauhan, Aviran Halstuch, Gil Shalev and Alina Karabchevsky *

School of Electrical and Computer Engineering, Ben-Gurion University of the Negev,

Beer-Sheva 8410501, Israel

* Correspondence: alinak@bgu.ac.il

To trace the required optimal mesh size, we plotted a convergence plot. For this, we carried out a numerical study exploring the influence of fine and coarse mesh on improving the results. The computational time which accounts for mesh size, was also studied.

The following numerical model convergence test was performed with Academic licensed COMSOL Multiphysics software 5.6 according to the numerical model presented in the numerical simulation section 2.1 of a 6 µm diameter microcylinder surrounded by air medium with total dimensions (including PML) of 60 µm x 60 µm. The PML thickness is about 7 µm.

The convergence test represents an intensity cross-section for four different mesh sizes of the wavelength λ (a = integer, n = refractive index).

Incident light is a linearly polarized continuous plane wave with wavelength λ = 637 nm and an initial intensity of 1 mW/m^2.

Figure S1 shows the convergence test for four mesh sizes: λ/3n, λ/6n, λ/9n, and λ/18n at the cross-section of y=0.

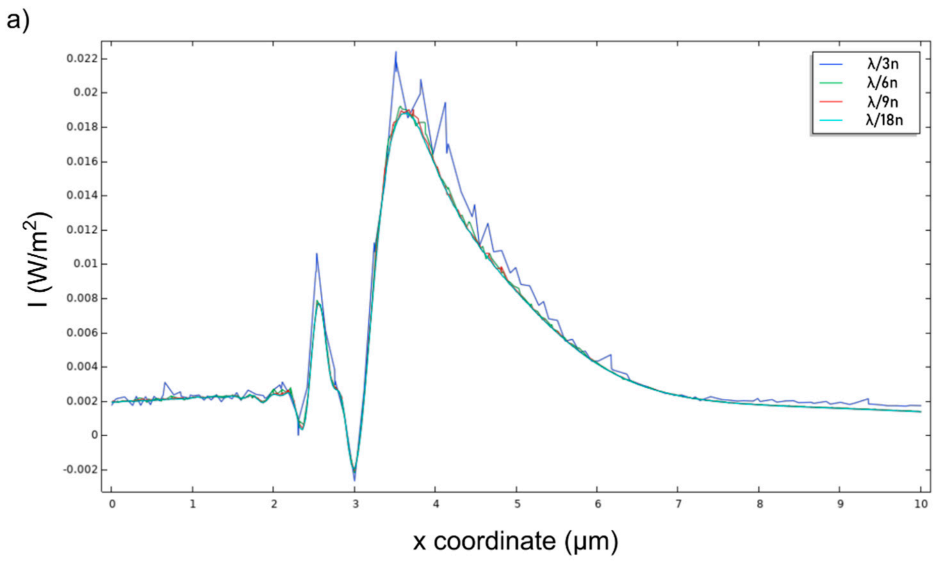

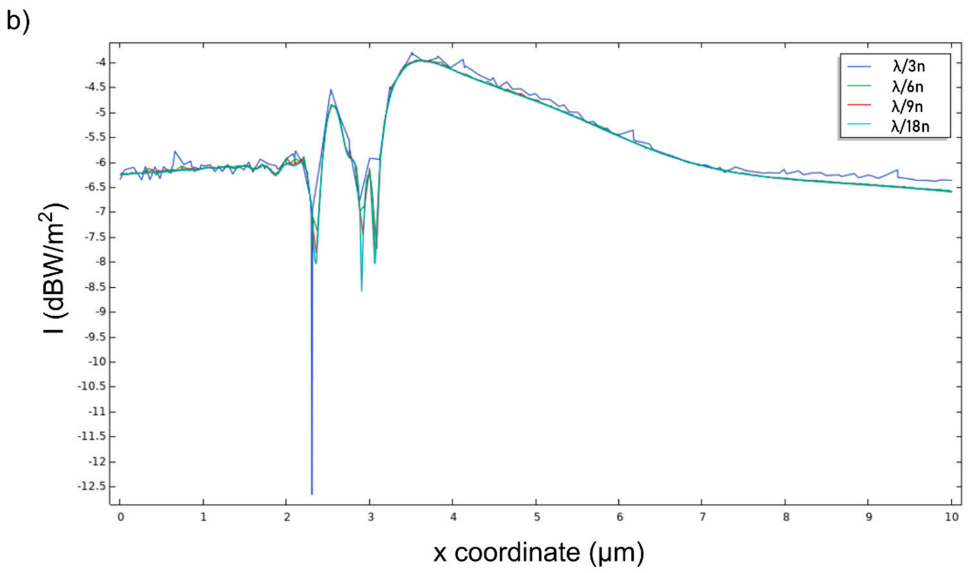

Figure S1: Numerical simulation convergence test at cross-section - convergence test for four mesh sizes: λ/3n, λ/6n, λ/9n and λ/18n at cross section of y=0. **a)** Intensity along the x-direction of the numerical model. **b)** A logarithmic view of intensity values.

According to the convergence plot in Figure S1, one can see that there are no significant differences between mesh size of λ/6n and λ/9n or λ/18n. Namely, the relative error between the obtained results is about 0.08%. The same relative error is observed when examining the full domain. Hence, it is justified to perform the numerical simulation with a mesh size of λ/6n for reliable and accurate enough results.

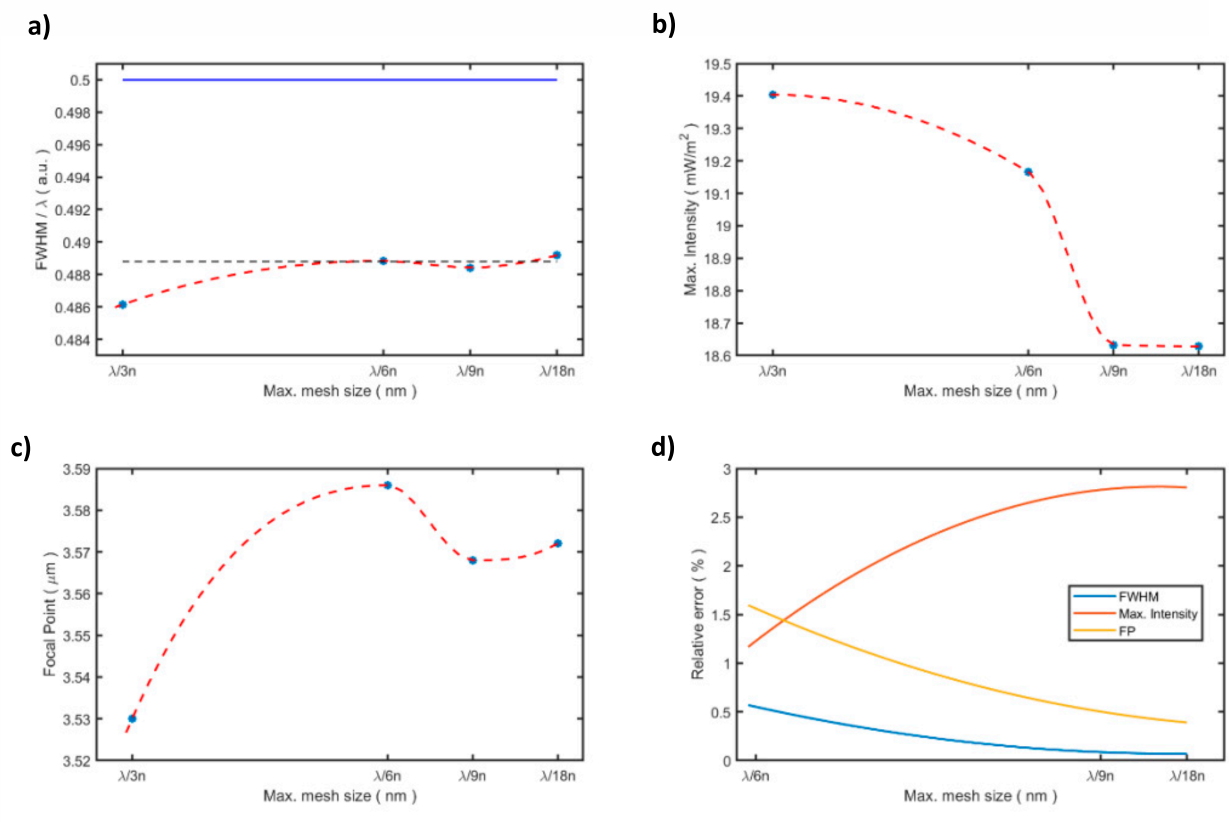

Figure S2: Calculated photonic nanojet characterized parameter as a function of 4 different mesh sizes: $\lambda/3n$, $\lambda/6n$, $\lambda/9n$ & $\lambda/18n$. **a)** FWHM in terms of incident wavelength (λ), **b)** Maximum intensity (mW/m^2), **c)** Focal point (F.P.) and **d)** relative error calculation between the mesh presented in the thesis and smaller mesh.

In Figure S2, one can see that the difference between the calculated FWHM with the mesh size of λ/6n (presented in the manuscript) and mesh that is three times smaller (e.g., $\lambda/18n$) is two orders of magnitude. Namely, the relative error between the obtained results is 0.08%. As for the calculated focal point and maximum intensity, the relative errors are 0.39% and 3.12%, respectively.
